# Analyzing PKC Gamma (+ 19,506 A/G) polymorphism as a promising genetic marker for HCV-induced hepatocellular carcinoma

**DOI:** 10.1186/s40364-022-00437-6

**Published:** 2022-11-30

**Authors:** Fizzah Abid, Talha Iqbal, Khushbukhat Khan, Yasmin Badshah, Janeen H Trembley, Naeem Mahmood Ashraf, Maria Shabbir, Tayyaba Afsar, Ali Almajwal, Suhail Razak

**Affiliations:** 1 Atta-Ur-Rahman School of Applied Biosciences, National University of Sciences and Technology, Islamabad, Pakistan; 2 Minneapolis VA Health Care System Research Service, Minneapolis, MN USA; 3 Department of Laboratory Medicine and Pathology, University of Minnesota, Minneapolis, MN USA; 4 Masonic Cancer Center, University of Minnesota, Minneapolis, MN USA; 5 School of Biochemistry and Biotechnology, University of the Punjab, Lahore, Pakistan; 6 Department of Community Health Sciences, College of Applied Medical Sciences, King Saud University, Riyadh, KSA Saudi Arabia

**Keywords:** PRKCG, K359R, Alanine aminotransferase, Biomarker, Serine-threonine kinases, Missense SNPs

## Abstract

**Background:**

HCC is a major health concern worldwide. PKC gamma, a member of the conventional PKC subclass, is involved in many cancer types, but the protein has received little attention in the context of single nucleotide polymorphisms and HCC. Therefore, the study aims to investigate the association of PKC gamma missense SNP with HCV-induced hepatocellular carcinoma.

**Methods:**

The PKC gamma nsSNPs were retrieved from the ENSEMBL genome browser and the deleterious nsSNPs were filtered out through involvingPredictSNP2, CADD, DANN, FATHMM, FunSeq2 and GWAVA. Among the filtered nsSNPs, nsSNP rs1331262028 was identified to be the most pathogenic one. Through involving I-TASSER, ProjectHOPE, I-Mutant, MUpro, mCSM, SDM, DynaMut and MutPred, the influence of SNP rs1331262028 on protein structure, function and stability was estimated. A molecular Dynamic simulation was run to determine the conformational changes in mutant protein structure compared to wild. The blood samples were collected for genotyping analysis and for assessing ALT levels in the blood.

**Results:**

The study identified for the first time an SNP (rs1331262028) of PRKCG to strongly decrease protein stability and induce HCC. The RMSD, RMSF, and Rg values of mutant and wild types found were significantly different. Based on OR and RR values of 5.194 and 2.287, respectively, genotype analysis revealed a higher correlation between the SNP homozygous wild Typeform, AA, and the disease while patients with genotype AG have higher viral load.

**Conclusion:**

Outcomes of the current study delineated PKC gamma SNP rs1331262028 as a genetic marker for HCV-induced HCC that could facilitate disease management after further validation.

**Supplementary Information:**

The online version contains supplementary material available at 10.1186/s40364-022-00437-6.

## Background

The human genome possesses various types of variation, and most abundant among these variations are SNPs (Single Nucleotide Polymorphisms). There are roughly 3–11 million SNPs, which compose almost 1% of the whole genomeCite ESM [1]. nsSNPs (non-synonymous SNPs) reside in the coding region and cause a change in the amino acid sequence, which may have neutral or deleterious effect on the protein [2–4]. Alteration in protein hydrophobicity, protein charge disturbance, change in protein geometry [5], altered stability, dynamics, translation and protein–protein interactions are as a result of nsSNPs [6–8]. GWAS(Genome Wide Association Studies) have identified thousands of SNPs in different genes including IL-28B, PNPLA3, KIF1B*, *PGD*, *UBE4B, MICA, and MDM2 that were associated with HCC (Hepaocellular Carcinoma) [9–12]. Poor prognosis in HCC is largely due to late diagnosis that renders traditional chemotherapy ineffective [13–16]. The SNPs and genetic variations have been identified as biomarkers [17]. Additionally, a lot of HCC-related biomarkers discovered so far are not sufficient to detect HCC at early stages and the detection is often missed, highlighting the need for novel biomarkers.

The protein kinase C gamma isoform belongs to the large family of PKC (Protein Kinase C) proteins. This enzyme is a serine/threonine-specific protein kinase, encoded by the PRKCG (Protein Kinase C Gamma type) gene located on chromosome 19 at position 19q13.2-q13.4 [18–21]. Moreover, the role of PKC gamma in the development and progression of cancer is well established [19]. Several studies have reported that PKC gamma is associated with cancer at different stages, i.e., glioma, kidney cancer, colon cancer and liver cancer [19]. However, all those studies established the role of PKC gamma in cancer progression and development. Its differential expression in various cancers does not provide sufficient information about the role of genetic polymorphism and its association with carcinogenesis. Missense SNPs in the PRKCG potentially affect its protein structure and function and thus may increase the likelihood or susceptibility of hepatocellular carcinoma development or progression. Moreover, knowing SNPs association with disease might improve the effectiveness of screening programs.

The aim of the study was to find the most deleterious variant in PRKCG gene through bioinformatics approach and determine the influence of that variant on the structure and function of the PKCγ protein. The study further aimed to determine the association between PRKCG variant (rs1331262028) and HCV-induced HCC. This research study has for the first time found a novel nsSNP (rs1331262028) within the family of cPKCs and proposes that the identified nsSNP can serve as a promising genetic marker mainly for HCV (Hepatitis C Virus)-induced HCC.

## Methods

### Data retrieval and structure prediction

**Fig. 1:**
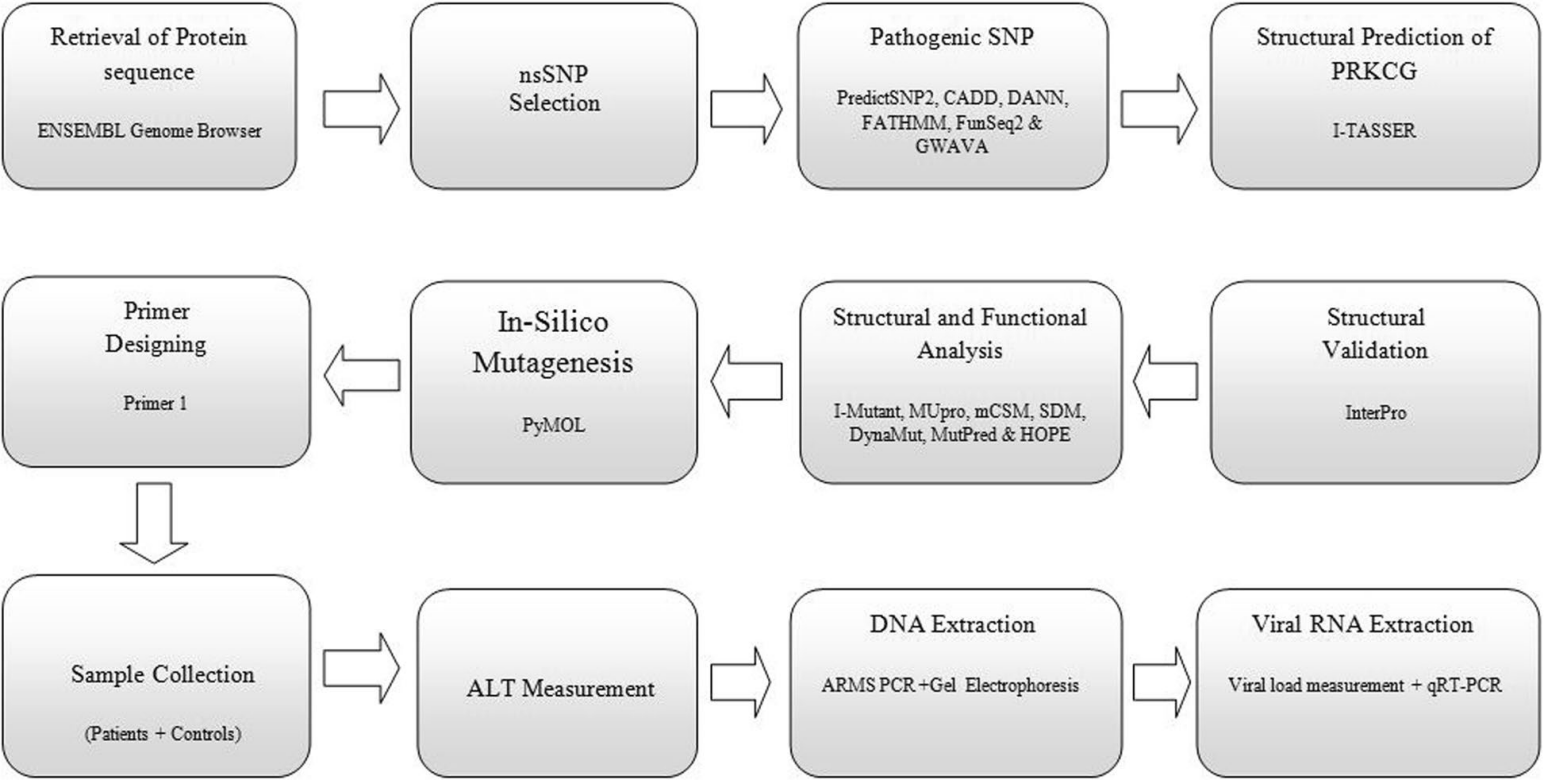
Workflow of methodology

The protein sequence of PKC gamma (ENST00000263431.4) was retrieved from ENSEMBL Genome Browser and missense SNPs were selected and retrieved from the variant table. These missense SNPs were then subjected to different toolsi.e, PredictSNP2, CADD, DANN, FATHMM, FunSeq2 and GWAVA to classify them as neutral or deleterious [22]. After further filtering, SNPs were sorted out that were predicted deleterious by all the six tools. Furthermore, InterPro [23] was used to determine the PKC gamma domains and their function. The selected nsSNPs were further analyzed to establish the impact of residue change on protein stability, structure and function. The amino acid sequence of the protein PKC gamma was submitted to I-TASSER [22] for structure remodeling and prediction. Among the five predicted model via I-TASSER, model 3 was selected based on C-score and InterPro analysis. The schematic representation of the study plan is shown in Fig. 1.

### Stability and structural analysis

Altered amino acid sequence may affect the stability of the protein. To analyze the effect of nsSNP on protein stability, five different web-based tools were used; i.e., I-Mutant [24], MUpro [25], DynaMut [26], mCSM [27] and SDM [27].The amino acid sequence in FASTA format was used as input for both I-Mutant and MUpro.ProjectHOPE [25] was also used, with the amino acid sequence of PKC gamma used as input. Substitution and amino acid positions were selected in the subsequent steps. A comprehensive report was generated at the end that provided information regarding impact of amino acid change on protein structure and hence its function. MutPred [25] was also used to predict the impact of amino acid change on protein structure and function.

### Molecular dynamic (MD) simulations

Molecular dynamic simulation for both wild type and mutated proteins was carried out via GROMACS software [24] to analyze the impact of residue change at different time scale in dynamic environment. Wild and mutated protein’s PDB (Program Database) were used as initial input in the simulation. The cubic box was generated and the system was solvated using water molecules and then neutralized with Na + /Cl ions.The original MD simulation's energy was minimized using steepest descent with a total of 50 000 steps, and then NVT and NPT equilibrium was achieved. After the simulation was run for both wild and mutated protein, the trajectory analyses command was entered (gmx_trjconv) followed by gmx_rms (for Root mean square deviations (RMSD) calculation), gmx_rmsf (for Root mean square fluctuations (RMSF) calculation),gmx_gyrate (for radius of gyration (Rg) calculation) and gmx_hbond (for number of hydrogen bonds calculation). The results obtained were then plotted graphically.

### Primer designing

The PCR (Polymerase Chain Reaction) primers were designed computationally via Primer1 [28]. The genome sequence mapped from chromosomal assembly 38. p13. was used as input in Primer1. SNP position and allele difference was selected, and the remaining options were kept as default.

### Genotyping and DNA Extraction

The collection of blood sample was approved from IRB (Institute Review Board), ASAB, NUSTand was conducted with the consent of the patients. The samples from a total of 100 HCV-induced HCC patients and 100 HCV negative controls were collected for genotyping analysis. For the inclusion criteria, only those patients that were HCV positive with confirmed HCC were included, while the controls that were HCV positive were excluded and only HCV negative patients i.e. healthy individuals were made the part of the study. All the HCV induced HCC patients were diagnosed at early stages. The information regarding the age and gender of patients and controls have been provided in Supplementary File 1, Table 1.

**Table 1:** List of different computational tools that were used to predict pathogenicity of nsSNP, their scoring criteria and classification

Tools	Score Range	Classification	
		**Neutral**	**Deleterious**
PredictSNP2	0–1	< 0.5	> 0.5
CADD	1–99	< 20	> 20
DANN	0–1	< 0.5	> 0.5
FATHMM	0–1	< 0.5	> 0.5
FunSeq2	0–1	< 0.5	> 0.5
GWAVA	0–1	< 0.5	> 0.5

The genomic DNA was extracted from all the samples using phenol–chloroform/organic method [29] and the DNA was then visualized through gel documentation system. Furthermore, ARMS-PCR (Amplification Refractory Mutation System-Polymerase Chain Reaction) was performed to detect single nucleotide change in PRKCG. Two sets of primers, 2 outer (forward with sequence 5' GGTAGGAGGGTGGCCA3' and reverse with sequence 5' CCGTCCCCTCAAGGAG 3') and 2 inner (forward with sequence 5' TTCCTCATGGTTCTAGGCAG 3' and reverse with sequence 5' ACCTTCCCAAAACTGCATT 3') were used. The inner primers were SNP specific and used for detection. The product size of the two outer primers was 476 and the product size of forward inner primer (G allele) was 224, while the product size for reverse inner primer (A allele) was 291.

### ALT (Alanine Aminotransferease) Test

The ALT test was performed for both patients and control samples to evaluate the effect of viral induced HCC on liver. The ALT kit was purchased from Merck (Darmstadt, Germany). Blood samples of both patients and control were collected in EDTA (Ethylene diamine tetraacetic acid)-vacationers’ tubes that were purchased from Becton, Dickinson and Company, USA. The manufacturer’s protocol of ALT kit was followed. The ALT concentration was checked through Microlab 300 semi-automated spectrophotometer at 340 nm.

### Viral RNA Extraction

To analyze the viral load in patient sample, viral RNA was extracted from the blood viaFavorPrep™ Viral DNA/RNA Kit (catalog no.FAVNK 001–1). 200 µl of blood was placed in an eppendorf tube and 500 µl of VEN buffer was added and vortexed for 5–7 s. 500 µl of 75% ethanol was added to the tube and again vortex was done for at least 5–7 s.The sample was transferred to the spin column and centrifuged at 8000 rpm for 1 min. The collecting tube was discarded, and the filter tube was put in a new tube. 500 µl of wash buffer 1 was then added and again centrifuged at 8000 rpm for 1 min. The collecting tube was changed and 750 µl of wash buffer 2 was added and centrifuged at 14000 rpm for 1 min. This step was repeated twice so the filter gets dried. Filter tube was transferred to Eppendorf tube and 50 µl of RNAase free water was added at the end and centrifuged again at 8000 rpm for 1 min. The RNA sample was transferredto tube and kept at -20 °C.

### Quantitative Reverse Transcriptase Polymerase Chain Reaction (qRT-PCR)

Real time qRT-PCR using SYBR green dye was used to simultaneously amplify and quantify viral RNA. For cDNA preparation, FIREScript® RT cDNA synthesis KIT was used that was purchased from SOLIS BIODYNE (Catalog number 06–15-0000S)0.6 µl of each viral sample, 43 µl of master mix and 1.2 µl of reverse transcriptase enzyme was mixed in single PCR tube. Two standards of known concentration, one lower and one upper limit, were used to determine the viral load of the samples. A plot was generated after the viral concentration was compared with standard curve, which provided the starting quantity of the template molecule on x-axis against the CT (Cycle Threshold) on Y-axis. The PCR conditions included initial denaturation at 95 °C for 5 min, annealing at 65 °C for 60 min, and extension at 72 °C for 10 min.

## Results

### Data processing and filtration

The ENSEMBL genome browser provided a total of 429 non-synonymous SNPs, which were then subjected to six different tools (PredictSNP2, CADD, DANN, FATHMM, FunSeq2 and GWAVA) to analyze their impact on protein structure and function. The nsSNPs were filtered that were predicted as pathogenic/deleterious by at least 3 to 4 bioinformatics tools based on scores (Table 1, Supplementary File 2, Table S2). After final filtering, 5 SNPs were sorted out that were predicted deleterious by all the six tools (Supplementary File 3, Table S3). rs1331262028 with genomic coordinates, + 19,506 A/Gwas selected because it has high pathogenicity scores, also it caused the change in the kinase domain of PKC gamma (K359R). The kinase domain of protein performs catalytic functions [17]. It has been known that SNPs located in kinase domain tends to increase the likelihood of cancer development [17].

### Effect of nsSNPon protein stability and structure

**Fig. 2:**
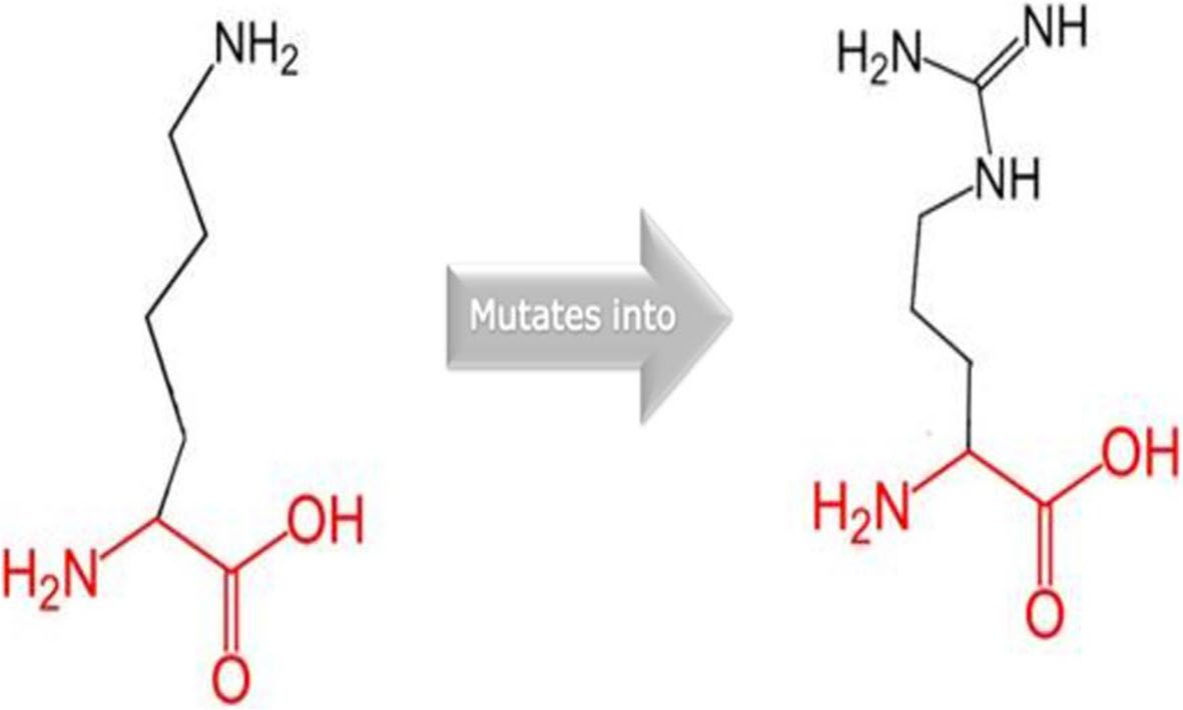
Schematic diagram of Residual change predicted in PKC gamma by Project HOPE. SNP rs1331262028 brings about substitution of arginine (left) in place of lysine (right). The size of arginine is larger from lysine because of two extra NH groups attachment

Stability analysis of PKC gamma (K359R) via five different *in-silico* tools (i.e., I-Mutant, MUpro, mCSM, SDM and DynaMut) predicted that this single amino acid substitution may alter protein stability and effect protein function. I Mutant, MUpro, mCSM and SDM predicted decreased protein stability with DDG values of -0.23, -0.11, -0.073 and -0.040 kcal/mol respectively, while DynaMut predicted increased protein stability due to the mutation with DDG value of 0.161 kcal/mol. The potential effects on protein structure and functions were analyzed by the two different tools, MutPred and HOPE. MutPred and ProjectHOPE demonstrated that amino acid change K359R (encoded by rs1331262028) can result in loss of protein kinase domain interaction with ATP (Adenosine triphosphate). Table 2 and Fig. 2 show changes in the stability, energy, and alteration of other protein functions upon mutation.

**Table 2:** List of possible alteration in the protein when lysine is changed with arginine at 359 position and their P-values estimated by MutPred2

Residual change	Mechanisms	*P*-Values
**K359R**	Loss of ubiquitination at K359	*P* = 0.027
	Gain of methylation at K359	*P* = 0.0351
	Gain of sheets	*P* = 0.0827
	Gain of phosphorylation at S361	0.0876
	Gain of MoRF binding	0.1603

### PKC gamma three-dimensional (3D) Structure Prediction

**Fig. 3:**
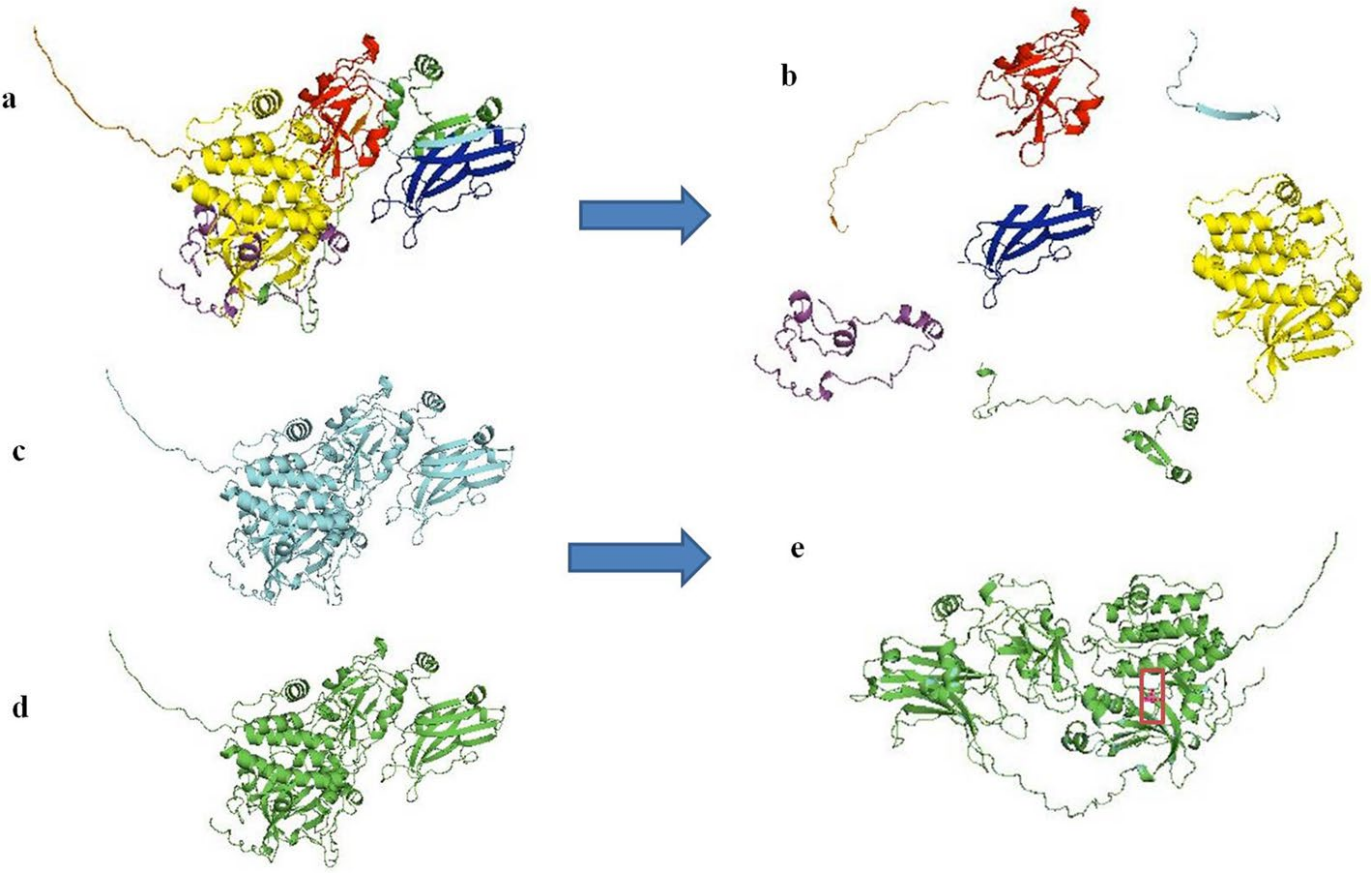
Tertiary structure of PKC gamma predicted through I-TASSER. **a** PKC gamma structure was selected based on C-score (-2.53). **b** Protein domains are highlighted in different colors, PS (Pseudosubtrate) (orange), PE/DAG-bd Domain(red color), (region between C1 and C2 domain (cyan), C2 domain (blue), Hinge region (green), Prot_kinase Domain (yellow) and AGC_kinase C Domain (magenta). **c** Wild PKC gamma structure **d** Mutated structure (K359R) **e** Aligned structure of Wild and Mutated structure

The 3-dimensional structure of PKC gamma was predicted via I-TASSER, which is advanced and reliable tool to predict protein structure based on multiple threading approach. I-TASSER predicted 5 different models. Among the five models, model 3 was selected because it has the lowest C-score (-2.53). The protein folding was then visualized through PyMol. The protein structure was computationally validated via InterPro, an online tool, by categorizing proteins in families and predicting different domains in the proteins. InterPro predicted four domains and other regions in the PKC gamma protein i.e.PE/DAG-bd Domain, C2 Domain, Prot_kinase Domain and AGC_kinase C Domain. These domains are shown in red, blue, yellow and magenta colors respectively. Also, PS (Pseudosubtrate), (region between C1 and C2 domain and Hinge region are shown (Fig. 3). Moreover, the Wild PKC gamma structure and mutated structure of PKC gamma structure (K359R) was aligned using PyMol (Fig. 3).

### Molecular Dynamics of PKC gamma

**Fig. 4:**
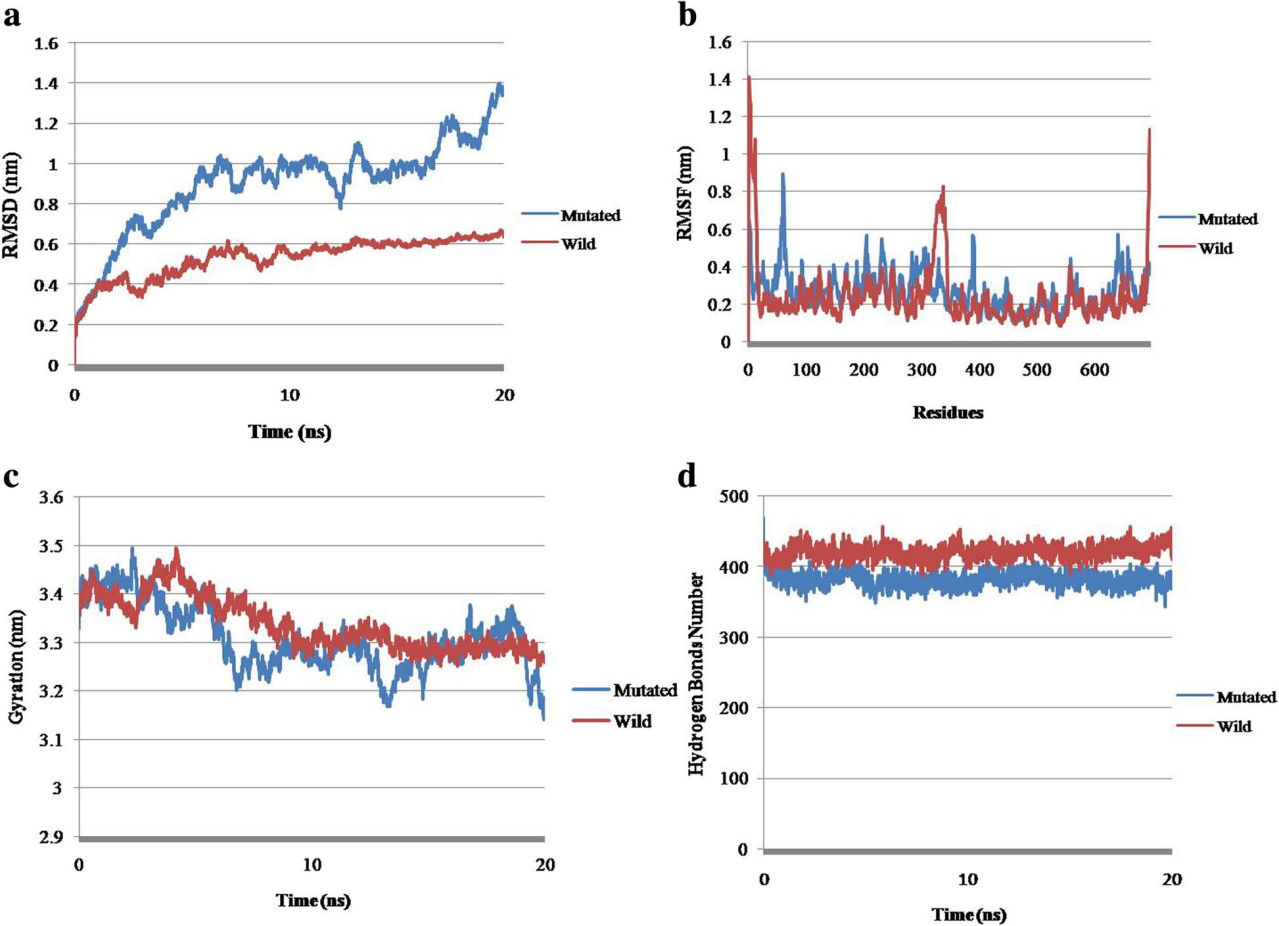
Graphical representation of MD simulation data. **a** RMSD graph representing the significance difference in the deviation pattern between wild type and mutated protein over time, **b** Amino acid residues of both wild type and mutated protein were plotted on x-axis against RMSF values on y-axis to analyze the difference in the fluctuation of both proteins from the reference point, **c** Radius of gyration of both wild type and mutated protein shows the compactness of protein in a dynamic setting as the simulation proceeds and **d** Represents the difference in the number of hydrogen bonds between wild type and mutated protein as simulation proceeds

After the simulations were run for both wild type and mutated PKC gamma structure, files were generated and data from those files were plotted on graphs to interpret simulation results. Four parameters were considered, i.e., RMSD, RMSF, radius of gyration and number of hydrogen bonds to analyze the difference in wild type and mutated protein. RMSD analysis revealed that the mutated protein deviates significantly from its reference position compared to wild type. When compared to the wild-type structure, the mutant protein displayed a gradually rising RMSD value with fluctuations (Fig. 4a). The highest RMSD value for mutant structure was recorded at 1.39 nm when the time had reached 19 ns. RMSF analysis showed the difference in the fluctuation of wild type and mutated protein residues. The region from 170–267 and 448–656 residues of the mutated protein had significant fluctuation from its mean point, which indicated that the protein structure expands over time. The comparison between wild type and mutated protein residue fluctuation is shown in Fig. 4b. The evaluation of radius of gyration calculated for both proteins showed a sharp increase in mutated protein peak up to 3.49 nm at 2.3 ns and gradual decrease to 3.3 nm as simulation run for 3.9 ns. From 6.8 to 12.9 nm, the gyration of mutated protein seemed to be at a steady phase, followed by gradual decrease to 3.1 nm as the simulation reached 13.3 ns. The radius of gyration of mutated protein remained stable to 19.2 ns, but at the end a significant decrease was shown. This data showed that the mutated protein loss its compactness at the start of simulation and became more compacted at the end. The significant difference in the two proteins Rg is represented in Fig. 4c. The hydrogen bonds number in mutated and wild type PKC gamma shows no significant difference as only a single amino acid was changed. The lines of both wild type and mutated proteins are overlapped over each other as shown in Fig. 4d that show there is no marked difference.

### Association of PRKCG SNP rs1331262028 with HCV associated HCC

For the analysis of the HCV associated HCC genotype data, the results showed high association of the SNP homozygous wildtype form (AA) with the disease as compared to homozygous GG and heterozygous AG genotypes based on OR and RR of 5.194 and 2.287, respectively, (*P*-value < 0.0001). The polymorphism in this allele may reduce the risk of disease occurrence. Table 3 shows the genotype data of patients and control.

**Table 3:** Association of PKCγ genotype (A/G) in patients and control

Genotype	Frequency Distribution		Odd Ratio		Relative Risk		*P*-Value
	**Patients**	**Control**	**Value**	**CI 95%**	**Value**	**CI 95%**	
AG	19%	33%	0.3827	0.2033 to 0.7236	0.5885	0.3882 to 0.8471	0.0046
GG	11%	31%	0.2751	0.1246 to 0.5777	0.4650	0.2675 to 0.7475	0.0008
AA	70.00%	31%	5.194	2.887 to 9.407	2.287	1.674 to 3.203	< 0.0001

### Association of PRKCG SNP rs1331262028 with gender in HCV associated HCC

The comparison of male and female patients with the controls replicate the results described in Table 2. The homozygous allele AA in both males and females was found to be associated with HCC. The OR (Odds Ratio) and RR (Relative Risk) for males was 6.021 and 2.310, respectively, and that of females was 4.737 (OR) and 2.291 (RR). The *P*-value for male and female having allele AA was < 0.0001 and 0.0002, respectively, emphasizing the significance of the results. The allelic data for gender is represented in Table 4.

**Table 4:** Association of PKCγ genotype (A/G) withgender

Genotype	Frequency Distribution		Odd Ratio		Relative Risk		*P*-Value
	**Patients**	**Control**	**Value**	**CI 95%**	**Value**	**CI 95%**	
AG (M)	24.00%	43.48%	0.4105	0.1768 to 0.9697	0.6316	0.3734 to 0.9871	0.0528
GG (M)	8.00%	30.43%	0.1988	0.06748 to 0.6852	0.3768	0.1503 to 0.7927	0.0078
AA (M)	68.00%	26.09%	6.021	2.551 to 14.71	2.310	1.527 to 3.657	< 0.0001
AG (F)	14.00%	33.33%	0.3256	0.1298 to 0.8953	0.5144	0.2558 to 0.9183	0.0237
GG (F)	14.00%	31.48%	0.3543	0.1408 to 0.9082	0.5426	0.2704 to 0.9627	0.0390
AA (F)	72.00%	35.19%	4.737	2.030 to 10.36	2.291	1.456 to 3.785	0.0002

### ALT enzyme level in Patient Vs Control

**Fig. 5:**
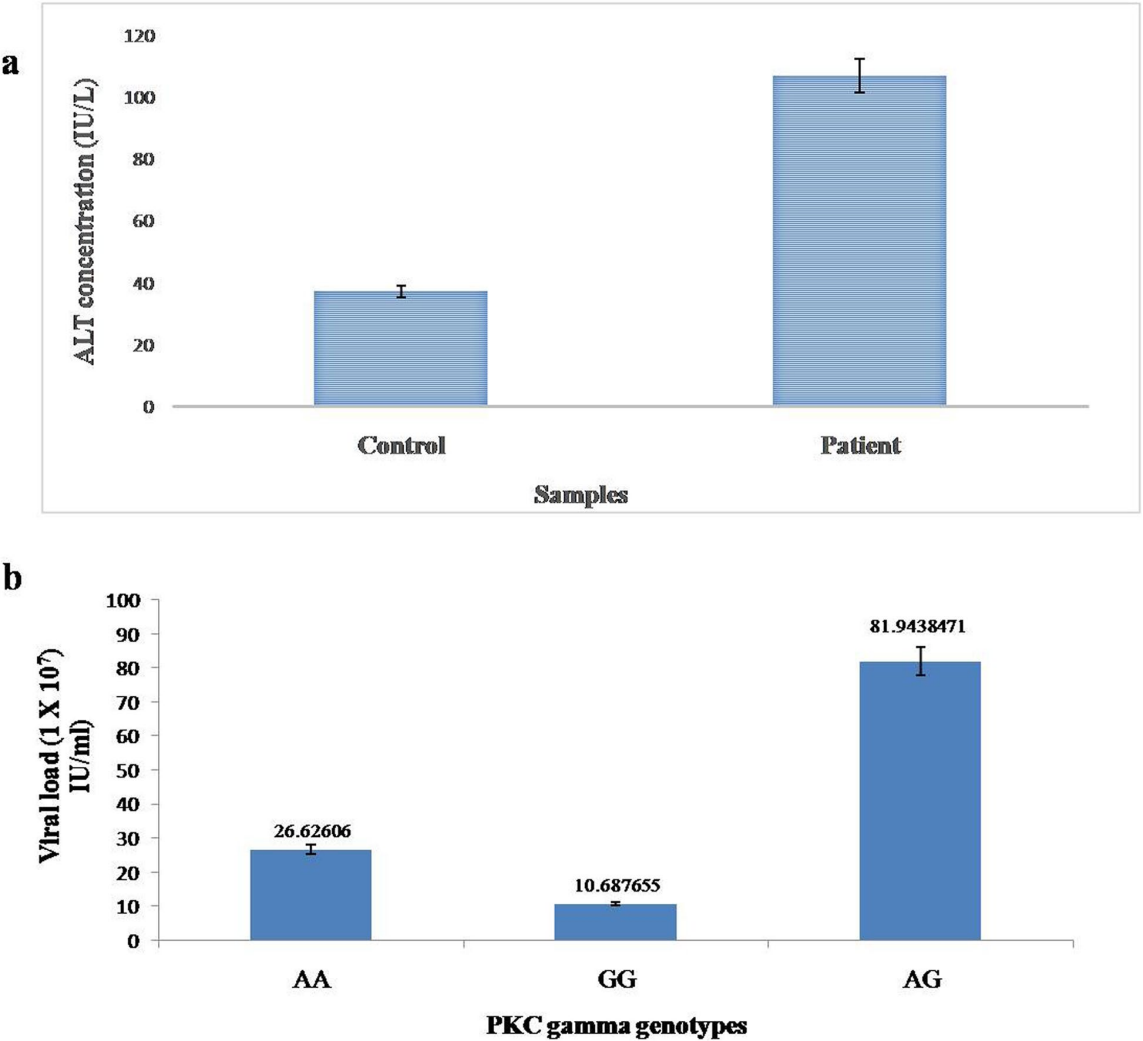
**a** Bar graph depicting the level of ALT in both patient and control. Mean concentration of ALT in patients were higher than healthy individuals. ALT concentration in both groups was measured in IU/L. **b** Bar graph representing the linkage of (A/G) genotype with viral load. Viral load was elevated in individuals harboring genotype AG in comparison to others. Least viral load among three genotypes was observed in genotype GG

The mean ALT levels of HCC patients were significantly higher when compared with the control samples. The average concentration of ALT in patients was 107 U/L, which was considerably higher from the normal ALT range of 7–40 U/L. The control sample showed mean ALT levels; under 40U/L. Figure 5a represents the comparison of ALT concentration in patient vs controls.

### Interplay of viral load with alleles of PKC gamma SNP rs1331262028 towards pathogenesis

Viral load was determined in HCC patient and control samples by qRT-PCR. The analysis of viral load against genotype showed a significant difference. The average viral load for genotypes AA, GG and AG was analyzed; and indicated that patients with AG allele had significantly high (819,438,471 copies/ml) viral load compared to patients with AA and GG alleles (Fig. 5b). These results suggest a potential correlation of viral load and genotype.

## Discussion

HCC is caused by several genetic and environmental factors. Among these, large proportion of HCC cases occurred because of viral infection [11]. HCV-mediated HCC prevalence is increasing globally specifically in developing countries [11]. The major problem in most cancers is the early detection. The SNPs affect different clinical parameters including staging, metastatic potential and treatment outcome [30, 31]. The studies indicate that the focal lesions (size and number) and portal vein invasion also correlate with gene polymorphisms [30]. So, the genotype association of PKC gamma with HCV induced HCC can be used as prognostic marker and studying SNPs can help indicate the detection of the disease in early stages and HCC is no more different. Therefore, the aim of this study was to investigate the association of PKC gamma missense SNP with HCV induced Hepatocellular carcinoma pathogenesis. PRKCG protein model predicted via I-TASSER belong to conventional PKC (cPKC) class that comprise of PKC alpha, PKC beta1 & 2 and PKC Gamma. The genotype association of PKC gamma with HCV induced HCC can be used as prognostic marker for the early diagnosis of the disease. The mutation in PKC gamma at position 359 from lysine to arginine falls within the kinase domain, which is present in all the members of conventional PKC [32]. As the two residues differ in size and charge, so it was estimated by Project HOPE that it might disturb the function of kinase domain of the protein considering the steric hindrance in that region. Additionally, protein mass and charge variations influence the spatiotemporal dynamics of interactions between proteins [17]. From MutPred, it was predicted that the mutation may cause loss of ubiquitination at K359 (*P* = 0.027); Gain of methylation at K359 (*P* = 0.0351); Gain of sheet (*P* = 0.0827); Gain of phosphorylation at S361 (*P* = 0.0876); Gain of MoRF binding (*P* = 0.1603). Moreover, it can be deduced that these changes could perturb the interactions with ATP as well. ATP interacts with the binding cleft/pocket of catalytic domain and mutation at that region notably affects and disconnects the favorable interactions [17]. Overall, all the predictions made by ProjectHOPE, I-Mutant, MUpro, mCSM SDM, DynaMut and MutPred in the study implicated that these changes expectedly lead to loss of thermodynamic stability. The I-TASSER model we chose was based on the C-score and InterPro prediction. It has been previously established that there is 40% similarity in protein sequence between cPKC and protein kinase A. I-TASSER has also been previously used in different studies to predict protein 3D model i.e., TAGAP, CCR6 and TOX3 [33–35]. The selection of I-TASSER was based on automated assessment of protein 3D structure prediction in CASP, which considered various parameters to confer accuracy of the predictor.

Molecular dynamic simulations also concluded the similar result that mutation at kinase domain is likely to make the protein less stable structurally. Also, higher fluctuations compared to wild structure were observed through RMSD, RMSF, Rg and hydrogen bond analysis. When compared to the original protein structure, our molecular dynamics technique revealed a shift of deviation in significant sections of the mutant structures that directly affects the secondary structure stability.

As the *in-silico* analysis estimated that mutation in PRKCG (K359R) may alter the structure and hence the function of the protein; so, to validate these prediction two sets of primers (two outer and two inner) were designed via Primer1 against rs1331262028 to find the correlation between allele change and association with hepatocellular carcinoma. The analysis of ARMS PCR results revealed that the wild type allele AA has strong correlation, OD (5.194), relative risk (2.287) and *P*-value > 0.0001, with HCC compared to homozygous GG and Heterozygous AG. The results analyzed based on gender and age group shows difference in OD and relative risk in male and female and suggest that a male with allele AA may have at higher risk compared to female, but no clear results were achieved from analysis based on age group because of the sample size. The association of polymorphism with genetic disease gave us an idea about susceptibility and can also be used for early diagnosis as the study of the osteoarthritis in Pakistani population revealed that polymorphisms in IL-6, TGF-beta-1 and CALM_1_ genes were associated with the disease [36]. It has been established that high ALT) levels are linked with HCV induced HCC) and can lead to the disease rapidly [37]. So, the ALT test was performed as a confirmatory test to differentiate patients and controls and the significant difference in ALT levels were detected in both the groups. The association of viral load with genotype was performed to analyze the link of genotype with viral load. Our results demonstrated that patients with genotype AG have high viral load followed by AA and then GG. Literature shows clearance of HCV viral RNA in patients co-infected with HCV/HIV having rs12979860 polymorphism CC genotype [38]. Further analysis of the PKC gamma in context of single nucleotide polymorphism requires investigating more nsSNPs and their association with HCV-induced HCC. The alteration in PKC gamma expression both at transcriptomic and proteomic level is also needed, which can be helpful regarding targeted therapy for HCC.

## Conclusion

In conclusion, the SNP identified may be used as a genetic marker, which can help us in the early diagnosis of the hepatocellular carcinoma. The expression profile of the PKC gamma upon this mutation needs to be explored, which may open new ways in the cancer therapeutic field and targeted drug therapy. The study findings emphasize the need for genome association studies and extensive clinical trial-based investigations on a broad population, so that the effect of the studied SNP could be studied extensively.  

## Supplementary Information

**Additional file 1:** **Supplementary Table S1.** Genotype data of all paticipants from both pateints and control group. HCC SAMPLES. **Table S2.** Pathogenic nsSNPs based on six different tools. **Table S3.** Filtered nsSNPs after applying filters.

## Data Availability

Data as supplementary material is provided along with the manuscript. Raw data will be available from corresponding author on request.
