# Supplementary material for: Analyzing PKC Gamma (+ 19,506 A/G) polymorphism as a promising genetic marker for HCV-induced hepatocellular carcinoma
Source: Biomark Res. 2022 Nov 30;10:87. doi: 10.1186/s40364-022-00437-6 (PMC9714225; doi:10.1186/s40364-022-00437-6)
Supplement: Supplementary file 1 — Additional file 1: Supplementary Table S1. Genotype data of all paticipants from both pateints and control group. HCC SAMPLES. Table S2. Pathogenic nsSNPs based on six different tools. Table S3. Filtered nsSNPs after applying filters. [file 40364_2022_437_MOESM1_ESM.pdf]

Analyzing PKC Gamma (+19506 A/G) Polymorphism as a Promising Genetic Marker for HCV-induced Hepatocellular Carcinoma

Fizzah Abid[1], Talha Iqbal[1], Khushbukhat Khan[1], Yasmin Badshah[1], Janeen H, Trembley[2,3,4], Kanza Shahid[1], Sameen Zafar[1], Amna Hafeez[1], Naila Khan[1],Naeem Mahmood Ashraf[5], Maria Shabbir[1*], Tayyaba Afsar[6], Ali Almajwal[6], Nawaf W. Alruwaili[6], Suhail Razak[6*].

Table S2: Pathogenic nsSNPs based on six different tools

Variant	PredictSNP2	CADD	DANN	FATHMM	FunSeq2	GWAVA
19:53882640, G→C	Deleterious	Deleterious	Deleterious	Deleterious	Deleterious	Deleterious
19: 53882648, T→A	Neutral	Deleterious	Neutral	Neutral	Deleterious	Deleterious
19: 53889693, G→A	Deleterious	Deleterious	Deleterious	Neutral	Deleterious	Deleterious
19: 53889719, G→A	Deleterious	Deleterious	Deleterious	Deleterious	Deleterious	Deleterious
19: 53889734, G→C	Deleterious	Deleterious	Neutral	Deleterious	Deleterious	Nil
19: 53889743, T→A	Deleterious	Deleterious	Deleterious	Deleterious	Deleterious	Nil
19: 53889743, T→C	Deleterious	Deleterious	Deleterious	Deleterious	Deleterious	Nil
19: 53889901, T→A	Neutral	Deleterious	Neutral	Neutral	Deleterious	Deleterious
19: 53889936, T→C	Deleterious	Deleterious	Deleterious	Deleterious	Deleterious	Nil
19: 53889963, G→A	Deleterious	Deleterious	Deleterious	Neutral	Deleterious	Nil
19: 53889963, G→T	Deleterious	Deleterious	Deleterious	Deleterious	Deleterious	Nil
19: 53898095, A→G	Deleterious	Deleterious	Deleterious	Deleterious	Deleterious	Deleterious
19: 53898097, G→A	Deleterious	Deleterious	Deleterious	Deleterious	Deleterious	Deleterious
19: 53900612, G→T	Deleterious	Deleterious	Neutral	Deleterious	Deleterious	Deleterious
19: 53906342, G→A	Deleterious	Deleterious	Deleterious	Deleterious	Deleterious	Deleterious
19: 53906729, T→C	Neutral	Deleterious	Deleterious	Neutral	Deleterious	Nil

Table S3: Filtered nsSNPs after applying filters

**Variants**	**PredictSNP2**	**CADD**	**DANN**	**FATHMM**	**FunSeq2**	**GWAVA**
19:53882640, G→C	Deleterious	Deleterious	Deleterious	Deleterious	Deleterious	Deleterious
19:53889719, G→A	Deleterious	Deleterious	Deleterious	Deleterious	Deleterious	Deleterious
19:53898095, A→G	Deleterious	Deleterious	Deleterious	Deleterious	Deleterious	Deleterious
19: 53898097, G→A	Deleterious	Deleterious	Deleterious	Deleterious	Deleterious	Deleterious
19:53906342, G→A	Deleterious	Deleterious	Deleterious	Deleterious	Deleterious	Deleterious

Supplementary Table S1: Genotype data of all paticipants from both pat

HCC SAMPLES

Age	Gender	viral load	ALT	AA	GG	AG
44	M	847539383	102	+	-	-
42	F	61700	92	+	-	-
38	M	39000	74	+	-	-
51	M	673000	85	+	-	-
27	M	847579493	117	+	-	-
52	F	8485748	94	+	-	-
24	F	8857749	169	-	-	+
43	M	948585730	66	+	-	-
32	M	39485580	91	+	-	-
55	F	9488843	75	+	-	-
43	M	394984478	95	+	-	-
46	F	8373723	61	+	-	-
58	M	921874654	125	-	-	+
35	F	83746339	66	+	-	-
32	F	8384743	67	-	-	+
35	F	938374	69	+	-	-
63	F	7293837	103	+	-	-
29	M	3943857403	193	-	-	+
32	M	847583930	183	+	-	-
38	M	74637839	75	+	-	-
50	M	836465493	78	+	-	-
50	M	93848745	81	+	-	-
40	F	9384750	81	+	-	-
29	F	3738493	84	+	-	-
25	F	8484883	84	+	-	-
35	F	9382746	88	-	-	+
27	M	833202938	172	-	-	+
52	F	347593	92	+	-	-
43	M	821939475	173	-	-	+
14	M	624403938	92	+	-	-
32	M	83747338	145	+	-	-
42	F	657448	210	+	-	-
42	M	475730200	153	-	-	+
43	M	938475653	104	-	-	+
29	F	364908323	129	+	-	-
72	M	7439298437	132	+	-	-
25	F	83949202	98	-	-	+
46	F	843292034	103	+	-	-
35	M	747362392	106	-	+	-
26	M	73298473	92	+	-	-
45	F	87000	94	+	-	-

55	M	39400	110	+	-	-
42	M	73700	72	-	-	+
36	F	3543110	14	+	-	-
32	F	74332282	57	+	-	-
32	M	736231	91	-	-	+
45	F	262000	87	-	+	-
55	M	246000	79	+	-	-
39	F	893700	62	+	-	-
30	M	1360000	97	+	-	-
55	M	72109832	153	-	-	+
31	F	63228289	94	+	-	-
26	F	7464939	73	+	-	-
30	F	79000	82	+	-	-
49	F	7438282922	110	-	-	+
38	F	873290109	96	+	-	-
33	M	978000	132	+	-	-
40	F	73218943	89	-	+	-
35	F	8587584	90	+	-	-
42	F	9484844	78	+	-	-
34	F	9484843	104	+	-	-
49	F	1179300	97	+	-	-
30	F	63922304	127	-	+	-
34	M	948757483	149	+	-	-
25	F	67333929	89	-	+	-
52	M	38380024	124	+	-	-
36	M	89000345	392	+	-	-
32	F	7439202	138	-	+	-
32	F	890073	97	+	-	-
39	M	7320922	64	+	-	-
47	M	119740	31	+	-	-
23	M	1167390	84	+	-	-
50	M	73020103	87	+	-	-
58	M	643000	56	+	-	-
48	M	493807	69	-	-	+
33	M	92721	45	+	-	-
59	F	1643000	98	-	-	+
37	F	84700	61	-	-	+
46	M	84878493	109	-	+	-
40	M	9488494	93	-	-	+
50	M	49000	220	+	-	-
40	M	63939434	126	-	+	-
45	F	283843186	210	+	-	-
52	M	48844833	82	+	-	-
49	M	94944432	98	+	-	-

23	F	21000	149	+	-	-
41	F	746363832	71	+	-	-
35	F	300000	48	-	+	-
40	M	189000	51	+	-	-
38	M	763500	179	-	-	+
34	F	73000	93	+	-	-
58	M	66337489	57	-	+	-
32	F	737338839	132	+	-	-
40	M	970000	95	+	-	-
45	F	78300	162	+	-	-
32	F	4646737	179	+	-	-
50	M	113276	291	+	-	-
45	F	647900	92	-	+	-
40	F	76300	115	+	-	-
36	F	5647747	61	+	-	-

eints and control group.

CONTROLS

Age	Gender	ALT	AA	GG	AG
51	M	35	-	+	-
27	M	41	-	+	-
52	F	27	+	-	-
24	F	29	-	+	-
43	M	36	-	+	-
32	M	45	-	+	-
55	F	23	+	-	-
43	M	37	-	-	+
46	F	57	-	+	-
43	M	28	-	+	-
35	F	49	-	+	-
32	F	38	-	+	-
35	F	34	-	+	-
63	F	39	-	+	-
29	M	35	-	+	-
32	M	35	-	-	+
38	M	30	+	-	-
50	M	41	-	-	+
40	F	35	-	-	+
42	F	41	-	+	-
25	F	25	-	+	-
35	F	37	-	+	-
27	M	29	-	+	-
25	F	41	+	-	-
43	M	44	-	+	-
14	M	38	-	-	+
32	M	51	-	-	+
42	F	45	+	-	-
43	M	37	-	-	+
29	F	43	-	-	+
53	M	47	-	+	-
25	F	57	-	+	-
46	F	39	-	+	-
35	M	16	-	+	-
26	M	32	-	+	-
45	F	49	-	+	-
42	M	51	-	+	-
36	F	45	+	-	-
32	F	91	+	-	-
55	M	29	-	-	+
39	F	38	-	-	+

65	M	29	+	-	-
55	M	40	+	-	-
42	F	29	+	-	-
26	F	36	-	-	+
49	F	45	-	-	+
38	F	23	-	+	-
33	M	37	+	-	-
40	F	57	-	+	-
35	F	28	-	-	+
86	F	49	-	-	+
34	F	38	-	-	+
49	F	34	-	+	-
30	F	39	-	-	+
34	M	35	-	-	+
73	F	35	-	-	+
52	M	30	-	-	+
36	M	41	-	-	+
32	F	35	-	-	+
62	F	41	-	-	+
39	M	29	+	-	-
47	F	36	-	-	+
23	M	45	-	-	+
50	M	23	+	-	-
74	F	37	-	-	+
64	M	57	-	-	+
33	M	28	+	-	-
59	F	49	+	-	-
46	M	38	+	-	-
40	M	34	-	-	+
50	M	39	+	-	-
40	M	35	-	-	+
45	F	35	+	-	-
52	M	30	-	-	+
49	M	41	-	-	+
23	F	18	+	-	-
41	F	28	-	-	+
35	F	32	+	-	-
40	M	31	-	-	+
38	M	33	-	+	-
34	F	29	-	-	+
58	M	31	-	+	-
32	F	31	-	-	+
32	F	32	-	-	+
50	M	33	-	-	+

45	F	32	+	-	-
40	F	30	+	-	-
36	F	29	+	-	-
44	M	30	+	-	-
42	F	33	+	-	-
38	M	26	-	-	+
50	F	26	-	+	-
42	M	31	-	-	+
55	F	57	-	+	-
32	M	37	+	-	-
45	F	53	+	-	-
30	F	41	+	-	-
37	F	39	+	-	-
40	M	27	+	-	-
45	F	40	+	-	-
